# 4D phase contrast MRI in intracranial aneurysms: a comparison with patient-specific computational fluid dynamics with temporal and spatial velocity boundary conditions as measured with 3D phase contrast MRI

**DOI:** 10.1186/1532-429X-14-S1-W3

**Published:** 2012-02-01

**Authors:** Pim van Ooij, Joppe Schneiders, Henk Marquering, Charles B Majoie, Ed vanBavel, Aart Nederveen

**Affiliations:** 1 Radiology, Academic Medical Center, Amsterdam, Netherlands; 2 Biomedical Engineering & Physics, Academic Medical Center, Amsterdam, Netherlands

## Background

It is believed that hemodynamic factors contribute significantly to aneurysm formation, growth and rupture. Studies attempting to predict risk factors are mostly based on computational fluid dynamics (CFD). A disadvantage of CFD is that among other assumptions, often non-patient-specific inflow conditions are prescribed. 4D phase contrast MRI (4D PCMRI) for the assessment of hemodynamic features may be preferred. In this study high resolution 4D PCMRI measurements in intracranial aneurysms are presented and compared with patient-specific CFD simulations in which a spatial and temporal velocity profile as measured with through-plane PCMRI in three directions (3D PCMRI) is prescribed as inflow boundary conditions.

## Methods

Retrospective gated PCMRI measurements were performed on a 3T MR system (Philips Medical Systems, Best, The Netherlands) in an 8-channel head coil in 4 patients. The slice for 3D PCMRI was planned perpendicularly to the artery proximal to the aneurysm. Scan parameters 3D PCMRI: 0.62x0.62x3 mm^3^; FOV: 200x200x20; TE/TR: 5.7/8.5 ms; FA: 10°; Cardiac phases: ± 36. Scan parameters 4D PCMRI: 0.8x0.8x0.8 mm^3^; TE/TR: 3.0/5.8 ms; FA: 15°; Cardiac phases: 10. Both scans used a Venc of 100x100x100 cm/s and a SENSE factor of 3. The lumen in both scans was segmented for all cardiac phases and in every slice of the fast field echo images using a level set evolution algorithm. The 3D PCMRI slice was registered onto the time of flight (TOF) geometry of the aneurysm. The TOF was subsequently registered onto the CDF mesh obtained from 3DRA datasets. The 3D PCMRI velocity information was interpolated to the faces of the CFD inflow boundary. CFD was performed using FLUENT (Ansys, Canonsburg, PA, USA), with density 1060 kg/m^3^ and viscosity 0.004 Pa.s. For a voxel-wise comparison between the 4D PCMRI and the CFD results, the CFD velocity information was registered and interpolated to the 4D PCMRI data.

## Results

**Figure 1:**
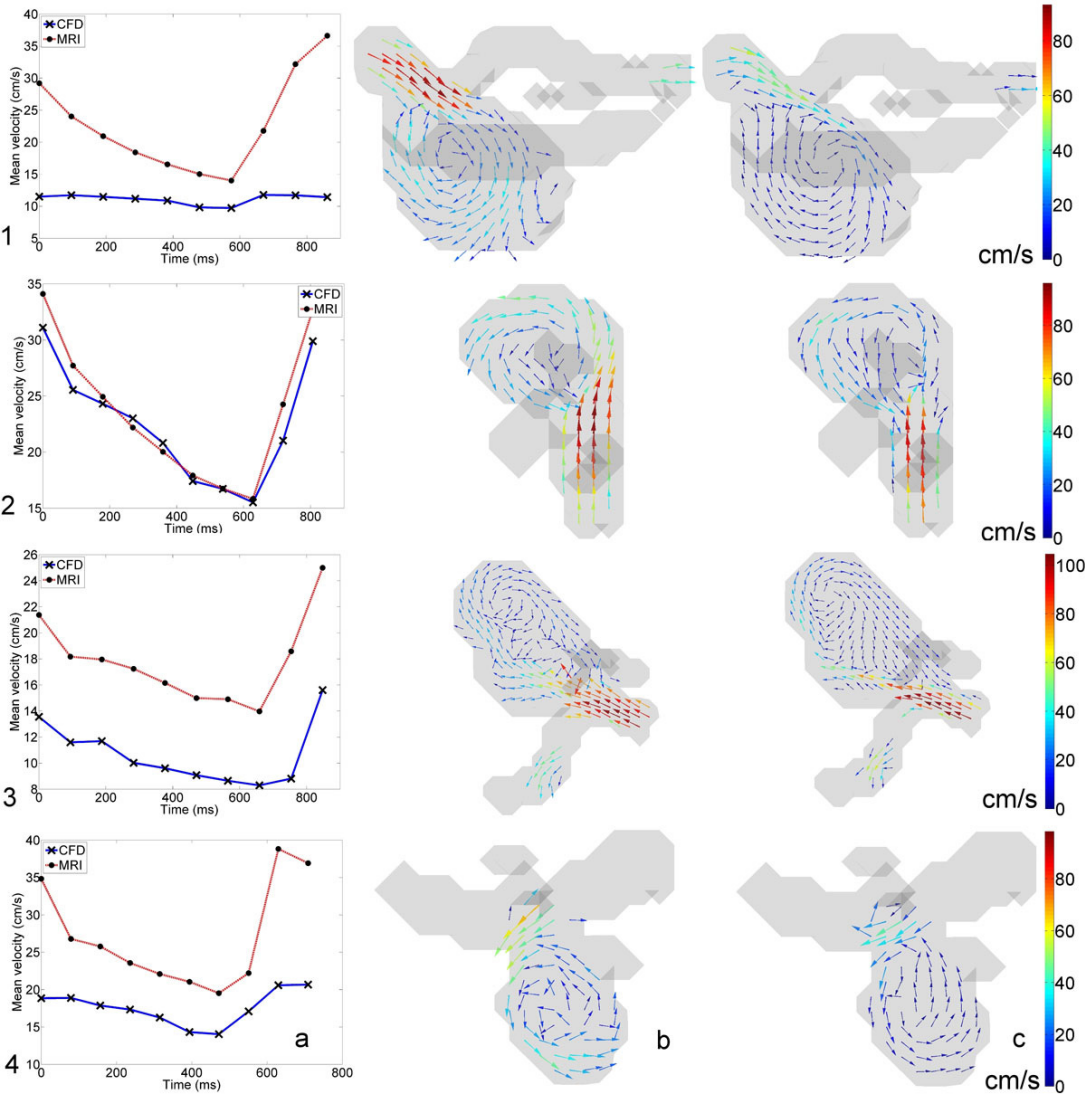
(a) Mean velocity MRI and CFD, (b) Velocity vectors MRI, (c) Velocity vectors CFD

The mean velocity in aneurysm 2 corresponded well, whereas the mean velocity in the CFD simulation was significantly lower for aneurysm 1, 3 and 4, see figure 1a. This is supported in table 1. The standard deviations were similar for all aneurysms. Qualitative similarities between PCMRI and CFD can be appreciated for all aneurysms, see figure 1b and c. High and low velocities are observed in similar regions as well as the main vortices. This is supported by similar median angles for all aneurysms in table 1.

**Table 1:** Location and size of the aneurysms, mean and standard deviation of the paired difference and median angle between velocity vectors as determined by MRI and CFD of the aneurysms shown in figure 1.

Location and size (length,width,height)	1. Left Middle Cerebral Artery (13.1 x 7.6 x 8.1)	2. Basilar Artery (8.7 x 6.3 x 7.4)	3. Right Middle Cerebral Artery (14.7 x 8.1 x 9.6)	4. Right Middle Cerebral Artery (7.21x 5.4 x 6.3)
Mean (cm/s)	11.6* (p=0)	1.6* (p=0)	7.0* (p=0)	10.5* (p=0)
SDp (cm/s)	12.7	12.8	10.0	13.2
Median angle (°)	22.8	23.6	36.3	22.4

## Conclusions

Higher mean velocities in PCMRI may be attributed to noise in the measurements and possible discrepancies in viscosity between the simulations and measurements. The 3D PCMRI of aneurysm 1 was performed in a different session than the 4D PCMRI. A disadvantage of CFD is the use of a static geometric vascular model, while PCMRI is segmented at each cardiac phase taking pulsatility into account. In CFD, however, the resolution is higher, and shows more flow details. Therefore, CFD and 4D PCMRI complement each other.

